# Non-Thermal Atmospheric Plasma Enhances Biological Effects of Fluoride on Oral Biofilms

**DOI:** 10.3390/jfb16040132

**Published:** 2025-04-05

**Authors:** Anushri Warang, Isha Deol, Sarah Fakher, Linfeng Wu, Liang Hong, Shaoping Zhang, Qingsong Yu, Hongmin Sun

**Affiliations:** 1 Department of Internal Medicine, University of Missouri, Columbia, MO 65211, USA; anushri.warang@health.missouri.edu (A.W.); sarah.fakher9@gmail.com (S.F.); 2 Department of Chemical and Biomedical Engineering, University of Missouri, Columbia, MO 65211, USA; ishadeol@missouri.edu; 3 Department of Public Health Sciences, College of Dentistry, Texas A&M University, Dallas, TX 75246, USA; linfengw@tamu.edu; 4 Department of Periodontics, College of Dentistry, University of Iowa, Iowa City, IA 52242, USA; shaoping-zhang@uiowa.edu; 5 Department of Mechanical and Aerospace Engineering, University of Missouri, Columbia, MO 65211, USA; yuq@missouri.edu

**Keywords:** fluoride, biofilms, *Streptococcus mutans*, *Streptococcus sanguinis*, dental caries, non-thermal atmospheric plasma

## Abstract

The objective of this study was an assessment of the anti-biofilm properties of fluoride non-thermal atmospheric plasma (FNTAP) generated using argon and hydrocarbon fluoride gas 1,1,1,2-tetrafluoroethane (TFE). These properties were evaluated by measuring the destruction and recovery of in vitro dual-species biofilms of *Streptococcus mutans* and *Streptococcus sanguinis* exposed to FNTAP at 5 or 10 standard cubic centimeters per minute (sccm) or argon non-thermal atmospheric plasma (ArNTAP) for 1 or 2 min, using resazurin-based reagent viability assays, colony forming units (CFU), culture media pH and live/dead staining. Both ArNTAP and FNTAP resulted in significant immediate reductions in bacterial load as compared to the control. Although ArNTAP did not significantly reduce biofilm regrowth, FNTAP treatment showed a bacterial load reduction of more than 5 log units of biofilm regrowth. FNTAP treatments significantly reduced the acidification of the culture medium after recovery incubation, indicating reduced living bacteria, with a pH of 6.92 ± 0.02 and 6.90 ± 0.03, respectively, for the 5 sccm and 10 sccm FNTAP treatments, as compared to a pH of 5.83 ± 0.26 for the ArNTAP treatment, and a significantly acidic pH of 4.76 ± 0.04 for the no-treatment groups. Our results suggest that FNTAP has exceptional anti-biofilm effects, and future directions of our research include the assessment of potential applications of FNTAP in clinical settings.

## 1. Introduction

Dental caries is a bacterial infectious disease that destroys tooth hard tissues. Although largely preventable, dental caries is the most common uncontrolled chronic disease worldwide [1]. Untreated caries in permanent teeth is the most prevalent among the 291 diseases and conditions assessed in the 2010 Global Burden of Disease Study [2,3].

Fluoride use in various forms has been the first line of defense against dental caries since the 1940s. Fluoride remains the most effective anti-caries agent and still forms the cornerstone for modern caries prevention [4,5,6]. The primary mechanism of action of fluoride in preventing caries is a topical effect through inhibiting the demineralization of tooth hard tissues and enhancing remineralization via the deposition of calcium fluoride and the incremental incorporation of fluoride in existing apatite in tooth mineral structures [6,7,8]. Its antimicrobial effects on oral bacteria and biofilms are considered secondary [9,10]. Fluoride use is recognized as responsible for the substantial decline in caries prevalence since the 1950s; however, its impact seems to have reached a plateau in recent decades. Fluoride affects the biological activities of cariogenic bacteria through carbohydrate metabolism [11], acid formation [12], intracellular pH regulation [13], extracellular polysaccharide production [14], and genomics or epigenomics [15]. However, its effects on oral bacteria and biofilms are much less understood. Most studies reporting the effects of fluoride on microorganisms have focused on individual bacterial species, and there have been few studies on its broad impact on oral microbial biofilms [15,16]. The effects of fluoride on microbial composition are reported to be minor [17,18]. More recently, increased fluoride resistance in microbial communities has become a concern [19,20,21]. Fluoride-resistant strains of *Streptococcus mutans* (*S. mutans*) have been isolated [22,23], and their phenotype and genetic changes including specific genes and their regulations have been studied [24,25,26,27,28,29]. Strategies have been actively sought to enhance the biological effects of fluoride on oral biofilm, including approaches to increase the fluoride sensitivity of oral bacteria [30,31,32,33].

Non-thermal atmospheric plasma (NTAP) is a non-equilibrium plasma generated at atmospheric pressure. NTAP contains highly reactive particles, including electronically excited atoms, molecules, ions, and free radical species [34], and reactive oxygen/nitrogen species. NTAPs have been studied for the purposes of sterilization [35,36], cancer treatment [37,38], wound healing [39,40], tissue regeneration [41,42,43], and delivering drugs [44].

The literature has attested that NTAP is an effective and efficient method for the destruction/disinfection of single-species oral bacteria and biofilms [45,46,47,48,49]. We previously reported that exposure to argon NTAP for less than 15 s could result in a 99.9999% reduction in living cell number for planktonic *S. mutans* seeded on various support media, including filter paper, glass slide, and polytetrafluoroethylene film [47]. We reported that exposure to argon NTAP for 2 min could achieve about 90% bacterial reduction for in vitro 3-day *S. mutans* biofilms grown on autoclaved, sintered, circular ceramic hydroxyapatite discs as determined by a 3-(4,5-Dimethyl-2-thiazolyl)-2,5-diphenyl-2H-tetrazolium bromide (MTT) assay [48]. Our studies also revealed that the recovery of *S. mutans* biofilms treated with Ar/O_2_ NTAP for 2 min was reduced by 70% relative to untreated control biofilms, as evaluated by a crystal violet staining assay [45].

The chemical composition of an NTAP is highly dependent on the plasma feed gas composition and the operating parameters. Our recent study [50] demonstrated that fluoride NTAP (FNTAP) generated using argon and hydrocarbon fluoride gas 1,1,1,2-tetrafluoroethane (TFE) could successfully deliver fluoride to enamel and effectively enhance the remineralization of human enamel in vitro.

In this study, FNTAP was further explored as an innovative approach to increase the fluoride sensitivity of oral biofilms, and we evaluated the effects of FNTAP generated with argon and TFE on in vitro dual-species oral biofilms of *S. mutans* and *Streptococcus sanguinis* (*S. sanguinis*).

## 2. Materials and Methods

### 2.1. Non-Thermal Atmospheric Plasma

NTAPs were generated using a custom-made atmospheric plasma brush device as described elsewhere [51], using argon gas (Industry Grade, Airgas, Radnor Township, PA, USA) as the plasma operating gas at a flow rate of 3000 standard cubic centimeters per minute (sccm) with or without TFE addition. The NTAPs were operated using a direct current (DC) mode by setting the DC value at 6.0 mA or 6.8 mA with a Spellman HV power supply SL60 (Spellman, New York, NY, USA). For generating FNTAPs, 5 sccm or 10 sccm TFE was added into the plasma feed gas in addition to argon. The argon and TFE feed rates were controlled by two separate mass flow controllers (MKS Instruments Inc., Andover, MA, USA). In this study, the above plasma parameters were selected to keep the plasm brush tip temperature close to the human body temperature of ~37.0 °C. The plasma brush tip temperature of NTAPs was ≤38.3 °C, measured using a grounded thermocouple (TJ36-CPSS-116G-3-SMPW-M, OMEGA Engineering Inc., Norwalk, CT 06854, USA). When adding 5 or 10 sccm TFE, the plasma brush tip temperature decreased a little. The plasma emission spectra of FNTAPs were recorded using an optical emission spectroscopy (OES) unit, Acton 2750 (Princeton Instruments, Trenton, NJ, USA).

### 2.2. Biofilm Culture

*S. mutans* (ATCC 700610) and *S. sanguinis* (ATCC 10556) were cultured separately in brain heart infusion (BHI) broth (Sigma-Aldrich, St. Louis, MO, USA) at 37 °C under 5% CO_2_ overnight. Optical density at 600 nm (OD600) was measured for the resulting bacterial suspensions and OD600 for *S. mutans* was adjusted by dilution with BHI broth to ensure that the two bacterial suspensions had similar OD600. Bacteria were harvested via centrifugation and resuspended in fresh BHI broth. Equal volumes of the resuspended cultures for both bacteria were mixed to form dual-species bacterial seeding suspension, which was supplemented with 0.5% sucrose. Artificial saliva (Pickering Laboratories, Mountain View, CA, USA) was used to coat the 24-well microplates used for biofilm formation. A total of 300 µL artificial saliva was added to each well of the 24-well microplates to coat the well bottoms at 4 °C overnight. Then, 1 mL of dual-species bacterial seeding suspension was added into each well of 24-well polystyrene microplates after the artificial saliva was removed from the wells, without allowing the wells to dry out completely. The seeded microplates were incubated at 37 °C under 5% CO_2_ overnight to form in vitro dual-species biofilms on the well bottoms. After the overnight incubation, the resulting in vitro biofilms were washed twice using 500 µL of Dulbecco’s Phosphate-Buffered Saline (DPBS) (Thermo Fisher, Waltham, MA, USA) for 3 min on a shaker before being used for the following experiments.

### 2.3. Assessing Immediate Destruction of In Vitro Dual-Species Biofilms by FNTAP

The washed in vitro dual-species biofilms were divided into 7 groups, including a negative control group which received no plasma treatment (No treatment group); two groups which were exposed to argon NTAP for 1 min (Ar 1 min group) or 2 min (Ar 2 min group), respectively; two groups which were exposed to FNTAP generated by adding 5 sccm TFE to argon for 1 min (TFE 5sccm 1 min group) or 2 min (TFE 5sccm 2 min group), respectively; and two groups which were exposed to FNTAP generated by adding 10 sccm TFE to argon for 1 (TFE 10 sccm 1 min group) or 2 min (TFE 10 sccm 2 min group), respectively. The plasma treatment time of 1 or 2 min was selected to accommodate clinically relevant treatment times, with <4.0 min treatment times being preferred. The biofilms in each group were treated according to their group assignment after removing the culture broth and washing with DPBS. Afterward, a PrestoBlue^TM^ (Invitrogen, Waltham, MA, USA) assay, live/dead staining assay, and CFU assays were conducted to assess the immediate effects of plasma on dual-species biofilms as described below (Section 2.5, Section 2.6 and Section 2.7).

### 2.4. Assessing Recovery of In Vitro Dual-Species Biofilms Exposed to FNTAP

The washed in vitro dual-species biofilms were divided into 4 groups based on preliminary data observed from the immediate killing effects of FNTAP, including a negative control group which received no plasma treatment (No treatment group), one group exposed to argon NTAP for 2 min (Ar 2 min group), one group exposed to FNTAP generated by adding 5 sccm TFE to argon for 2 min (TFE 5 sccm 2 min group), and one group exposed to FNTAP generated by adding 10 sccm TFE to argon for 2 min (TFE 10 sccm 2 min group). The biofilms in each group were treated according to their group assignment after removing the culture broth. Following the treatments, the samples in each group were divided into 2 subgroups. For the first subgroup, 1 ml of fresh BHI broth supplemented with 0.5% sucrose was added to the each of the wells. In addition, for the second subgroup, 1 mL of fresh BHI broth supplemented with 0.5% sucrose and 0.02% hydrogen peroxide was added to each well. The addition of hydrogen peroxide was performed to assess the impact of oxidative stress on biofilm recovery based on our previous studies of other oral biofilms [45,52]. The 24-well microplates were incubated at 37 °C under 5% CO_2_ overnight to allow the treated biofilms to recover. Afterward, culture media pH measurement, PrestoBlue assay, live/dead staining assay, and CFU assays were conducted on the recovered dual-species biofilms as described below (Section 2.5, Section 2.6, Section 2.7 and Section 2.8).

### 2.5. PrestoBlue Assay

The PrestoBlue™ (Invitrogen, Waltham, MA, USA) assay was used to assess the viability of bacteria within the biofilms. PrestoBlue™ viability reagent is a ready-to-use, nontoxic, resazurin-based solution that serves as a cell health indicator for fluorescence and absorbance-based microplate assays. Biofilms were incubated with 300 µL of PrestoBlue^TM^ reagent for 30 min according to the manufacturer’s instructions. The optical density of 100 uL of reagent in each well was measured at 570 nm and 600 nm. The bacterial viability was calculated from the optical densities according to the manufacturer’s instructions.

### 2.6. Colony Forming Units (CFU) Assay

The total colony forming units (CFU) of *S. mutans* and *S. sanguinis* in biofilms were counted by plate counting on Mitis Salivarius Agar (MSA) (Sigma-Aldrich, St. Louis, MO, USA) plates supplemented with 1% potassium tellurite (Neogen, Lansing, MI, USA). The biofilm was scraped from the bottom of each well with autoclaved wooden dowels. The biofilm was suspended in 200 µL of the remnant PrestoBlue^TM^ reagent by pipetting up and down to form a homogenous suspension. Serial dilutions were made for all wells. The dilutions plated for the untreated control and argon NTAP groups were 10^−3^, 10^−4^ and 10^−5^. The dilutions plated for the FNTAP 1 min and 2 min groups were undiluted, 10^−1^ and 10^−2^. These dilutions were selected based on pilot experiments. All dilutions were spread on square MSA plates with grids to facilitate bacterial colony counting. Plates were incubated at 37 °C with 5% CO_2_ for 72 h, after which the CFU were counted.

### 2.7. Live/Dead Staining Assay

Treated and regrown biofilms were washed twice with DPBS for 3 min and stained using the LIVE/DEAD™ Cell Imaging Kit (488/570) (Invitrogen, Waltham, MA, USA) following the manufacturer’s instructions. Live Green (Comp. A, 1 mL 1 µM solution) was transferred into Dead Red (Comp. B, 1 µL lyophilized stock) and was added to the wells immediately. The plate was incubated at room temperature for 15 min. Kit components are optimized for the common green and red imaging filters—FITC (excitation/emission 488 nm/515 nm) and Texas Red (excitation/emission 570 nm/602 nm). The stained biofilms were examined after staining using a BioTek Lionheart FX Automated Microscope (Agilent Technologies, Santa Clara, CA, USA) and pictures were taken with red and green filters at 4× magnification superimposed and separately to record living and dead biofilm cells. Superimposed images were analyzed with the open-source image processing package Fiji (ImageJ, US National Institutes of Health, Bethesda, MD, USA) to quantify proportions of live/dead biofilm cells in the images. The red and green channels were split, and the fluorescence intensities were measured after subtracting background. The mean fluorescence intensity ± standard deviation obtained for each color was recorded.

### 2.8. pH Analysis

The pH values of the bacterial culture media were measured after biofilm recovery incubation to assess the effects of plasma treatment on the culture media acidification due to acids produced by living *S. mutans*. The pH of culture broth in each well was measured using an Orion™ ROSS Ultra™ (Thermo Fisher, Waltham, MA, USA) mini pH electrode.

### 2.9. Statistical Analysis

The relative viability of the treatment groups was assessed by dividing the viability of each treatment group well by the average viability of untreated control wells. CFU counts were log-transformed. The experiments for assessing immediate destruction of in vitro dual-species biofilms by FNTAP were repeated twice with triplicates for each group in one experiment so that each group included a total of 6 replicates (n = 6). The experiments for assessing recovery of in vitro dual-species biofilms exposed to FNTAP were repeated thrice with triplicates for each group in one experiment so that each group included a total of 9 replicates (n = 9). Numerical data for relative viability and CFU counts were analyzed with Graphpad Prism (GraphPad Software Inc., Boston, MA, USA) and reported as mean ± standard error. Student’s *t*-test was used to analyze the statistical difference between two groups.

## 3. Results

### 3.1. FNTAPs

**Figure 1:**
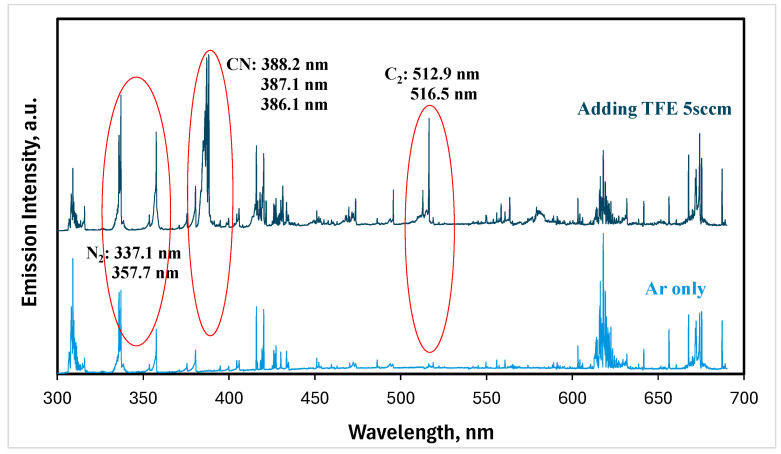
Typical optical emission spectra of argon NTAPs (Ar only) and FNTAPs (adding TFE 5 sccm). Other plasma parameters were 3000 sccm Ar, 6.0 mA DC, and 0.97 kV voltage.

The plasma brush tip temperature was ~38.3 °C for argon NTAPs. This plasma brush tip temperature decreased a little with the addition of TFE. The plasma chemistry of FNTAPs was characterized using optical emission spectroscopy (OES). A N_2_ second positive system was observed for argon NTAPs due to the N_2_ in air, in addition to the typical argon emission lines (Figure 1). Very strong optical emission of CN bands and C_2_ bands appeared in the spectra for FNTAP in addition to the emission lines observed for argon NTAP, as shown in Figure 1.

**Figure 2:**
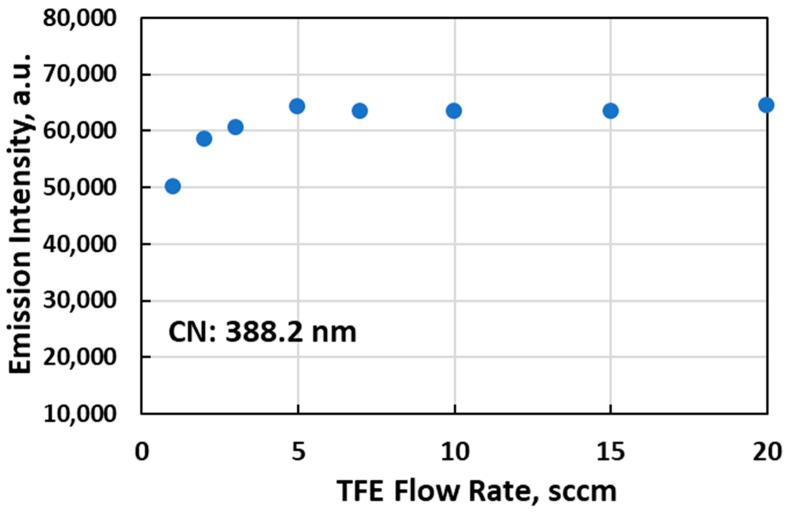
Change in CN optical emission intensity with TFE flow rates in FNATPs. Other plasma parameters were 3000 sccm Ar, 6.0 mA DC, and 0.97 kV voltage.

In this study, the F atom emission line was not directly detected with the strong emission of CN and C_2_ bands that appeared in the OES spectra of FNTAPs. However, since TFE is the only source of C atoms for FNTAP, the CN emission intensity should properly indicate the fragmentation of TFE molecules. More specifically, the C in CN came from the breakdown of TFE to F atoms and other hydrocarbon fluoride plasma species, thus releasing C atoms to form CN and C_2_ in FNTAPs, as detected in the OES spectra. CN emission indicated the fluoride-related plasma species including F atoms being present in the NTAPs. The CN emission intensities started leveling off at the addition of 5 sccm TFE among all FNTAP generated in this study (Figure 2). The CN emission intensity represents the concentration of CN and thus a quantitative indication of TFE fragmentation in FNTAPs. It is therefore determined that the addition of 5 and 10 sccm TFE was used for biofilm tests in this study. It should also be pointed out that the FNTAP OES spectra in Figure 1 and Figure 2 were collected under plasma conditions of 6.0 mA. Our OES results showed that the plasma current change from 6.0 mA to 10 mA only affected the optical emission intensity but did not change the optical emission characteristics. The plasma parameter of 6.8 mA, which produces much more stable plasmas than using 6.0 mA, was therefore used to generate FNTAPs for biofilm treatment in this study.

### 3.2. Immediate Effects of FNTAPs on S. mutans and S. sanguinis Dual-Species Biofilm

**Figure 3:**
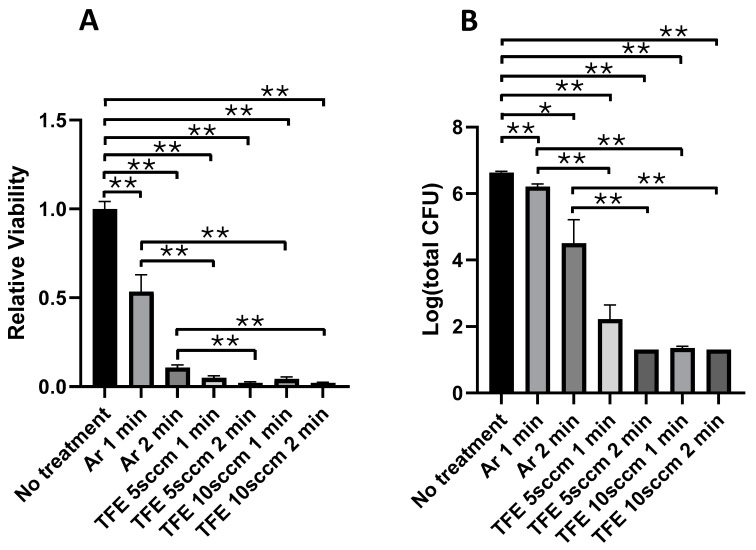
FNTAPs significantly reduced the bacterial load of *S. mutans*/*S. sanguinis* dual-species biofilms in vitro, as shown by PrestoBlue™ reagent analysis (**A**), and CFU count per well (**B**). The CFU counts per well in the TFE 5 sccm 2 min and TFE 10 sccm 1 and 2 min FNTAP treatment groups were below the detection level (<20), except for one data point for TFE 10 sccm 1 min, where the total CFU count was 40. The experiment was repeated twice with triplicates for each group in one experiment so that each group included a total of 6 replicates (n = 6). * *p* < 0.05, ** *p* < 0.01. Other plasma parameters were 3000 sccm Ar, 6.8 mA DC, and 1.00 kV voltage.

Figure 3 shows the bacterial survival in in vitro dual-species biofilms immediately after exposure to different NTAPs, including argon NTAP and FNTAPs for 1 or 2 min. Figure 3A summarizes the results of PrestoBlue^TM^ assays for the biofilms immediately after NTAP exposure. Argon plasma treatment resulted in a reduction in bacterial viability of 46.6 ± 10.4% (*p* < 0.01) and 89.3 ± 4.5% (*p* < 0.001) for the 1 and 2 min plasma exposures, respectively, as compared to the untreated control group. Exposure to FNTAP generated with argon plus 5 sccm TFE reduced the bacterial viability by 95.0 ± 4.3% and 97.9 ± 4.2% for 1 min and 2 min plasma exposure, respectively (*p* < 0.001), as compared to the untreated control group. Exposure to FNTAP generated with argon plus 10 sccm TFE resulted in a reduction in bacterial viability of 95.7 ± 4.3% and 98.0 ± 4.2% for 1 min and 2 min plasma exposure, respectively (*p* < 0.001), as compared to the untreated control group. Compared to NTAP generated with argon only, the addition of either 5 sccm TFE or 10 sccm TFE resulted in a statistically significant greater reduction in bacterial viability for both 1 and 2 min plasma exposures (*p* < 0.001). However, no significant statistical difference was observed between dual-species biofilms exposed to either of the two FNTAP treatments for the same duration of either 1 min or 2 min (*p* > 0.6).

Figure 3B summarizes the results of CFU assays for in vitro dual-species films immediately after the plasma exposure. A reduction of 0.42 ± 0.09 and 2.13 ± 0.72 log units of CFU was achieved by the argon plasma treatment with a treatment time of 1 and 2 min (*p* < 0.001, *p* < 0.02), respectively, as compared to the untreated control group. However, with the addition of 5 sccm TFE, a reduction of 4.41 ± 0.42 log units of CFU (*p* < 0.001) was achieved with 1 min plasma treatment as compared to the untreated control group. The CFU count in the 2 min FNTAP treatment group was below the detection level (<20). Increasing the TFE flowrate to 10 sccm achieved a CFU reduction to below the detection level (<20) for both the 1 and 2 min treatment groups, except for one data point for TFE 10 sccm 1 min, where the total CFU count per well was 40. The CFU count for the FNTAP treatment groups was significantly different compared to the argon NTAP treatment groups at both 1 min and 2 min plasma exposure times (*p* < 0.01). Of note, the CFU count of the no-treatment group was 6.63 ± 0.04 log units. These results are consistent with the results of viability assays for the in vitro dual-species biofilms immediately after plasma exposure.

**Figure 4:**
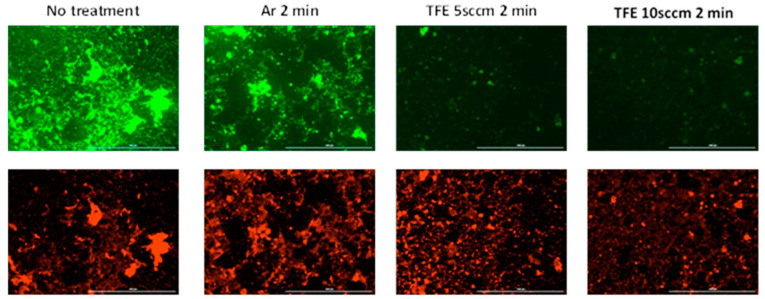
Fluorescence microscopy images of biofilms of no treatment, 2 min treatment with argon plasma, 2 min treatment with TFE 5 sccm FNTAP, and 2 min treatment with TFE 10 sccm FNTAP groups. Green color: living cells at 4× magnification. Red color: dead cells at 4× magnification. Other plasma parameters were 3000 sccm Ar, 6.8 mA DC, and 1.00 kV voltage. Scale bar: 1000 µm.

Figure 4 presents the fluorescence microscope images for the in vitro dual-species biofilms stained immediately after the plasma exposure. The immediate killing effects indicated that 2 min treatment resulted in the maximum bactericidal activity. Hence, we selected 2 min for live/dead assays. A red color represents dead bacterial cells, and a green color represents living bacterial cells (Figure 4). Both living and dead cells were observed in the biofilms receiving no plasma treatment. This is typical of living biofilms. There were markedly decreased live cells, as indicated by mean green fluorescence intensity, in the 2 min argon plasma-treated samples (22.97 ± 39.95) and 2 min FNTAP-treated samples (5.26 ± 15.23 for 5 sccm and 4.13 ± 11.01 for 10 sccm) as compared to the untreated control (43.21 ± 49.09), in agreement with the viability and CFU results. The mean red fluorescence intensity was similar across all groups, including the untreated control (34.28 ± 56.01). The live/dead proportion, as measured by mean green fluorescence intensity/mean red fluorescence intensity, was 1.26, 0.49, 0.13 and 0.13, respectively, for the control, argon NTAP 2 min, 5 sccm FNTAP 2 min and 10 sccm FNTAP 2 min groups. The direct imaging of biofilms provides valuable information about the distribution of live and dead bacteria in biofilms, which is not obtainable by viability and CFU count assays. However, there is little consensus about how to quantitatively evaluate biofilm images. Using the light intensity of fluorescent dyes to represent live/dead cells has limitations. The accuracy of the results may be compromised by nonspecific staining or extracellular matrix staining. Image analysis does not examine the entire biofilm. The quality of the images may also impact the results. Therefore, the results of the quantitative image analysis should be interpreted with caution. Nevertheless, our image analysis results are generally in agreement with the CFU and viability results, showing that there was a large reduction in live bacterial cells in the FNTAP-treated groups as compared to the untreated control and argon NTAP-treated groups.

### 3.3. Recovery of S. mutans and S. sanguinis Dual-Species Biofilms Exposed to FNTAP

**Figure 5:**
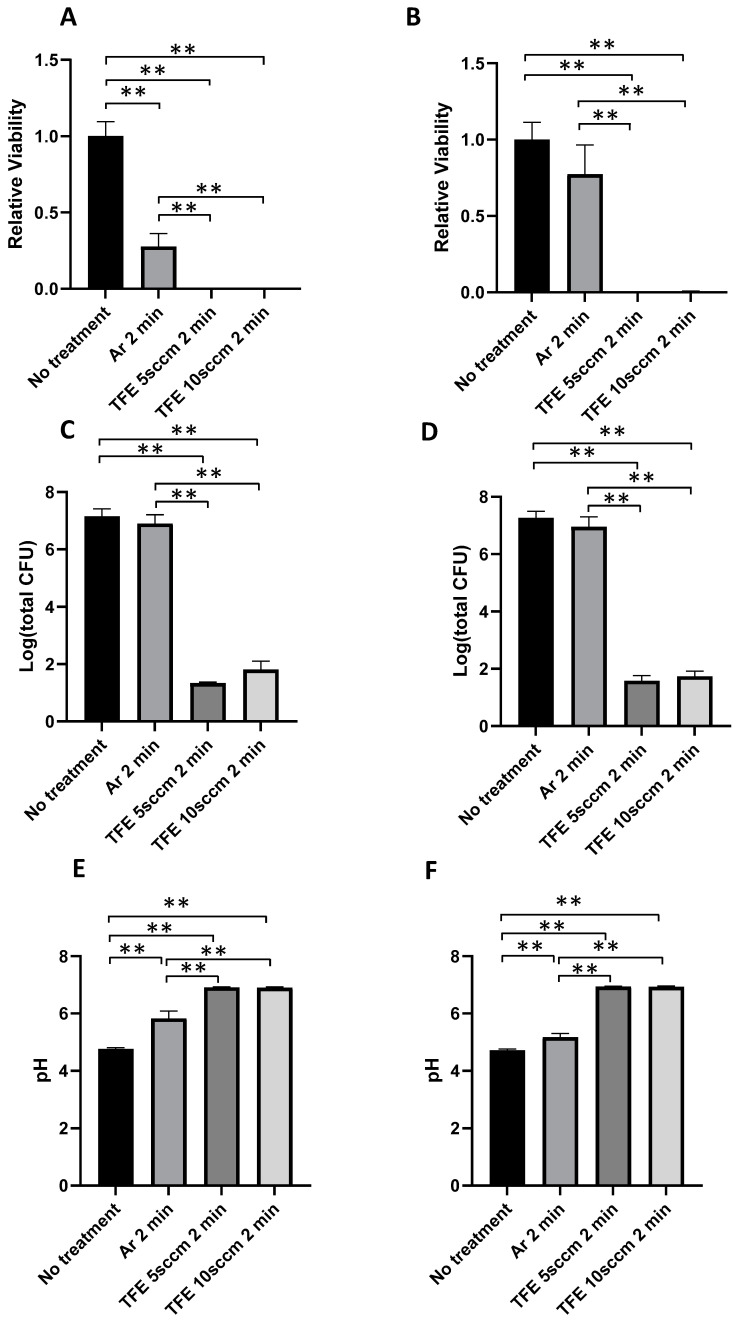
FNTAPs significantly inhibited the regrowth of *S. mutans*/*S. sanguinis* dual biofilms in vitro, as shown by PrestoBlue™ reagent analysis. Viability (**A**) without H_2_O_2_ during recovery and (**B**) with H_2_O_2_ during recovery. CFU counts per well (**C**) without H_2_O_2_ during recovery and (**D**) with H_2_O_2_ during recovery. pH value of the culture medium (**E**) without H_2_O_2_ during recovery and (**F**) with H_2_O_2_ during recovery. The experiment was repeated in triplicate so that each group included a total of 9 replicates (n = 9). ** *p* < 0.01. Other plasma parameters were 3000 sccm Ar, 6.8 mA DC, and 1.00 kV voltage.

The immediate killing effects indicated that the 2 min treatment resulted in the maximum bactericidal activity. Hence, we selected 2 min for the recovery assays. After the plasma exposure, dual-species biofilms were cultured in fresh BHI broth supplemented with 0.5% sucrose at 37 °C under 5% CO_2_ overnight for recovery with and without the addition of 0.02% H_2_O_2_. Figure 5A shows the bacterial viability in in vitro dual-species biofilms after the recovery incubation following plasma exposure for 2 min without H_2_O_2_ during the recovery incubation. A bacterial viability of 27.8 ± 8.3% was observed for the biofilms recovered after being exposed to argon NTAP for 2 min as compared with the 100 ± 9.6% bacterial viability for the recovered biofilms without plasma exposure (*p* < 0.001). Almost negligible bacterial viability was detected for the biofilm recovered after being exposed to both FNTAPs for 2 min with 0.10 ± 0.02% for FNTAP with 5 sccm TFE and 0.12 ± 0.03% for FNTAP with 10 sccm TFE (*p* < 0.001) (Figure 5A). There were also significant differences in bacterial recovery between the FNTAP-treated groups and argon NTAP-treated group (*p* < 0.01) (Figure 5A).

On the other hand, for the biofilms exposed to H_2_O_2_ during recovery, there was no significant difference in viabilities between the argon plasma-treated biofilms and control group (*p* > 0.3), though there was still a trend of lower viability in the argon-treated group. Almost negligible bacterial viability was detected for the biofilm recovered after being exposed to either FNTAP for 2 min with 0.26 ± 0.06% for FNTAP with 5 sccm TFE and 0.58 ± 0.23% for FNTAP with 10 sccm TFE (*p* < 0.001) (Figure 5B). Significant differences in bacterial recovery were observed between the FNTAP-treated groups and argon NTAP-treated group (*p* < 0.01) (Figure 5B).

Interestingly, after recovery incubation, the CFU counts for biofilms exposed to argon NTAP for 2 min were only slightly lower than those for biofilms recovered without plasma exposure. There was no statistically significant difference in CFU counts for these two groups (*p* > 0.4) (Figure 5C,D) regardless of whether they were exposed to H_2_O_2_ or not during the recovery, even though there was a statistically significant difference in bacteria viability (Figure 5A). Compared to argon NTAP, the addition of either 5 sccm TFE or 10 sccm TFE resulted in a statistically significant reduction in bacterial recovery (*p* < 0.001) (Figure 5C,D). There was a more than 5-log-unit reduction in CFU counts for the biofilm recovered after being exposed to either FNTAP for 2 min compared to the biofilms recovered after no plasma treatment (*p* < 0.001), regardless of H_2_O_2_ exposure during recovery (Figure 5C,D).

The pH of the culture media was measured at the end of the recovery incubation (Figure 5E,F). For biofilms recovered without H_2_O_2_ exposure, the culture media had an acidic pH of 4.76 ± 0.04 after no plasma exposure. A significantly higher pH of 5.83 ± 0.26 was observed for biofilms recovered after 2 min of argon NTAP exposure (*p* < 0.001). For biofilms exposed to FNTAP generated with 5 sccm and 10 sccm TFE, pH values of 6.92 ± 0.02 and 6.90 ± 0.03, respectively, were detected (*p* < 0.001), approaching a physiologically neutral range. Similar pH changes were observed in biofilms recovered under H_2_O_2_ exposure during recovery (Figure 5F). When biofilms were recovered in the presence of H_2_O_2_, the culture media had an acidic pH of 4.72 ± 0.04 after no plasma exposure. A significantly higher pH of 5.18 ± 0.13 was observed for biofilms recovered after 2 min of argon NTAP exposure (*p* < 0.01). For biofilms exposed to FNTAP generated with 5 sccm and 10 sccm TFE, pH values of 6.94 ± 0.02 and 6.94 ± 0.02, respectively, were detected (*p* < 0.001). There were also significant differences in pH values in FNTAP-treated groups in comparison with argon NTAP-treated groups (*p* < 0.001).

**Figure 6:**
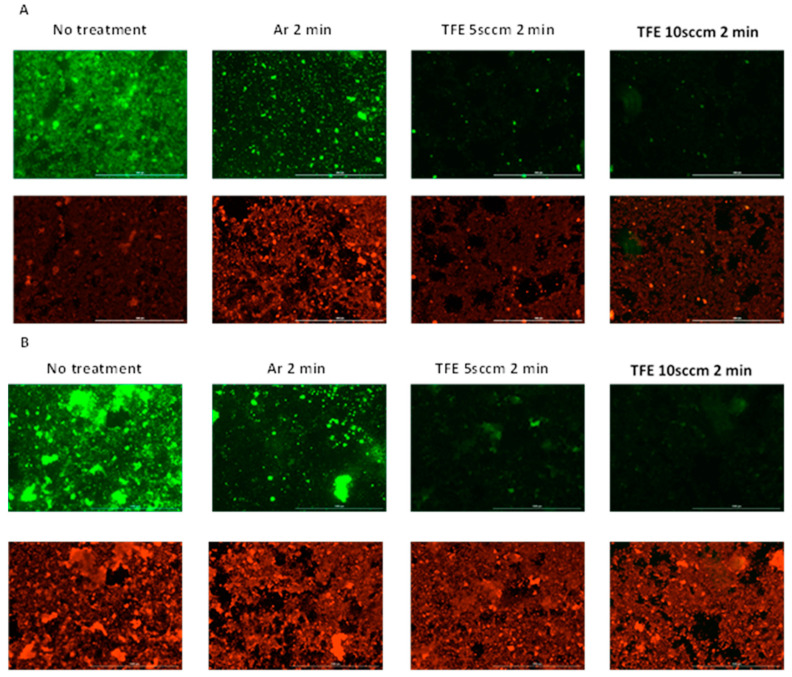
Fluorescence microscopy images of regrown biofilms after different treatments (no treatment, 2 min treatment with argon plasma, 2 min treatment with TFE 5sccm FNTAP, 2 min treatment with TFE 10 sccm FNTAP) without H_2_O_2_ during recovery (**A**) and with H_2_O_2_ during recovery (**B**). Green color: living bacteria. Red color: dead bacteria. Scale bar: 1000 µm.

The regrown biofilms were stained with a live/dead staining kit to observe the living and dead cells (Figure 6). Superimposed images of the live and dead cells were analyzed using the Fiji package. For biofilms without H_2_O_2_ exposure during recovery, there were both living and dead cells in the recovered biofilms in the no-treatment group (mean green fluorescence intensity 35.07 ± 28.36, mean red fluorescence intensity 23.52 ± 19.27). There were a lower number of living cells in the argon plasma-treated biofilms (mean green fluorescence intensity 18.61 ± 35.87). Fewer living bacteria were observed in the recovered biofilms exposed to either FNTAP for 2 min (mean green fluorescence intensity 4.70 ± 15.68 for 5 sccm and 3.87 ± 11.53 for 10 sccm), indicating a strong inhibition of biofilm regrowth after FNTAP treatment. Similar results were observed for subgroups with H_2_O_2_ exposure during recovery after each plasma treatment (mean green fluorescence intensity 43.77 ± 49.08 for untreated control, 19.23 ± 37.97 for argon plasma-treated, 8.34 ± 14.36 for 5 sccm FNTAP-treated and 9.73 ± 14.36 for 10 sccm FNTAP-treated groups). The live/dead proportion, as measured by the mean green fluorescence intensity/mean red fluorescence intensity, was 1.49, 0.35, 0.15 and 0.11, respectively, for the control, argon NTAP 2 min, 5 sccm FNTAP 2 min and 10 sccm FNTAP 2 min groups without H_2_O_2_ exposure and 0.74, 0.29, 0.14 and 0.15, respectively, for the control, argon NTAP 2 min, 5 sccm FNTAP 2 min and 10 sccm FNTAP 2 min groups with H_2_O_2_ exposure. These results are in agreement with the CFU and viability results in that there was a substantial reduction in recovered live cells in the FNTAP-treated groups in comparison with the untreated group and argon NTAP-treated group.

## 4. Discussion

While fluoride exhibits antimicrobial effects at high concentrations [53,54], fluoride resistance among oral bacteria has been reported and studied [19,20,21,22,23]. Research efforts have been devoted to developing strategies to improve the antimicrobial effects of fluoride. One major approach is to enhance the susceptibility of oral biofilm bacteria to fluoride [10]. In this study, we investigated the use of non-thermal plasma as a novel method for fluoride delivery. The results suggest that this approach significantly enhances the sensitivity of oral bacteria and biofilm to fluoride, achieving substantial improvements in effectiveness and demonstrating exceptional, robust anti-biofilm properties.

Our previous studies have shown that non-thermal argon plasma treatments can effectively disinfect planktonic bacteria including *S. mutans* on various surfaces [47,49,55], and also bacteria in caries-related biofilms [45,48]. However, no study has been reported for the antibacterial and anti-biofilm properties of FNTAP. The addition of hydrocarbon fluoride gas TFE to argon plasma in this study significantly enhanced the destruction and elimination of the dual-species biofilm by plasma. These results indicate that FNTAPs destroy the biofilms by effectively killing the bacteria in the dual-species biofilms.

The destruction efficiency of plasma on dual-species biofilms is related to the exposure time. Treating biofilms for longer times could markedly increase the killing effects in the argon, argon+TFE 5 sccm and argon+TFE 10 sccm treatment groups (Figure 3). The increase in plasma exposure time improved the killing of the biofilm bacteria. This was attributed to the fact that more bactericidal reactive species generated in the plasma interacted with the biofilms due to the long plasma exposure.

The addition of TFE in argon plasma could improve plasma destruction efficiencies on dual-species biofilms. The mechanisms of action of fluoride on oral bacteria and biofilm have been explored. Several potential pathways have been proposed and discussed, such as carbohydrate metabolism, acid formation, intracellular pH regulation, extracellular polysaccharide production, and genomic or epigenomic changes. A previous study [18] showed that 400 ppm and 1400 ppm sodium fluoride solutions did not affect the bacterial compositions and growth of oral biofilms consisting of *Actinomyces oris*, *Candida albicans*, *Fusobacterium nucleatum*, *Streptococcus oralis*, *Veillonella dispar* and *Streptococcus sobrinu*. However, the pH for the fluoride-treated group was less acidic with a value of 5.5 as compared with pH 4.5 for the untreated control. The authors attributed this observation to a decrease in acid production or extracellular polysaccharide (EPS) production for the fluoride-treated group. Fluoride-resistant *S. mutans* could produce more EPS and increase enzymatic activities and the pH in oral biofilm [20]. Our present study suggested that fluoride delivered through non-thermal plasma dramatically impacts the living bacterial numbers, metabolic activities, and pH production of dual-species biofilms and biofilm recovery after plasma treatment. The conventional mechanical treatment cannot completely remove the bacteria in biofilms, leading to the recolonization and regrowth of the biofilm after treatment. Bacterial regrowth will make the eradication of the regrown biofilm more difficult. Our previous studies have shown that NTAP treatment using argon or argon/O_2_ could slow down the regrowth of the treated biofilms under the stress of reactive oxygen species during recovery [45,52]. This stress of reactive oxygen species is often observed in the host immune response in controlling oral biofilms. In the present study, the recovery patterns of the biofilms were studied after plasma treatment. Although there was a significant reduction in biofilm CFU immediately after argon plasma treatment, the biofilm regrew after fresh medium was added, which had a slightly lower CFU count as compared with the no-treatment group. It was interesting that there was no significant difference between the CFU counts of the recovered biofilms treated by argon plasma and those with no treatment, even though there was a significant difference in viability when measured by the PrestoBlue™ assay. This might suggest that argon plasma treatment caused physiological changes in the recovered biofilm, which resulted in the bacteria having a lower level of metabolic activity than the no-treatment group. Our previous studies also showed that plasma treatment could change the metabolic activity of biofilms [45]. It seems that argon treatment only inhibits the biological activities of the biofilm, such as bacterial metabolism, but it is not able to destroy/kill resident bacteria in the biofilms. On the other hand, a significant reduction in regrowth was observed for FNTAP-treated biofilms using both 5 sccm and 10 sccm TFE plasma, with more than 5-log-unit-lower CFU counts than the control. Of note, in our previous reports, the inhibitory effects on biofilm recovery were more significant under oxidative stress via exposure to hydroperoxide, while a much less inhibitory effect on regrowth was observed without oxidative stress [45,52]. In the current study, the inhibitory effects of biofilm recovery were observed both with and without oxidative stress for FNTAP. This indicated that the addition of hydrocarbon fluoride gas TFE to argon NTAP exerted exceptionally potent anti-biofilm activity. Only a small number of bacteria survived the treatment, and the regrowth was significantly reduced without external oxidative stress.

*S. mutans* generates acids from carbohydrate fermentation and thrives under acidic conditions. As expected, no-treatment control group exhibited an acidic pH value. The argon plasma-treated group showed a higher pH value than the control, although there were similar CFU numbers. This suggested that argon plasma treatment inhibits acid production in dual-species biofilms. The FNTAP-treated groups exhibited pH values that were almost physiologically neutral, which could be due to there being few living bacteria in the biofilms after recovery, suggesting that FNTAP treatments could lead to a healthier oral microenvironment compared to no treatment. Compared to other strategies to enhance the biologic effects of fluoride on oral biofilms, which usually affect metabolic activities and pH production but do not change the bacterial numbers and composition in oral biofilms [18,56], our strategy of using non-thermal plasma to deliver fluoride could dramatically change both growth and metabolic activities.

The present study explored using non-thermal plasma to deliver fluoride and assessed the effects of fluoride plasma on some biological activities of dual-species oral biofilms. The results are very promising in terms of using fluoride plasma to control oral biofilms. However, some limitations must be acknowledged. This is the first study to report the effects of fluoride plasma specifically on oral biofilms. The overall sample sizes of each experiment were not very large, and the results need to be further validated in studies with larger sample sizes. This study only used in vitro, dual-species oral biofilm models. More advanced oral biofilm models and in vivo animal studies are needed to understand the effects in situations more closely mimicking real-life conditions. Also, only limited evaluation of metabolic activities and pH change was performed, and the mechanisms of action of fluoride plasma towards oral biofilms are largely unclear. It is possible that plasma and fluoride have synergetic effects or could produce new active plasma species, hypotheses which need further research.

## 5. Conclusions

The addition of TFE in argon plasmas significantly improved the disinfection efficiency on dual-species biofilms formed by *S. mutans* and *S. sanguinis*. The plasma disinfection efficiency on the dual-species biofilms also depended on the treatment time. Of particular interest, the FNTAP treatments were able to significantly inhibit the regrowth of the biofilms after treatment, while the argon plasma treatment largely failed to do so, indicating that adding TFE to plasma markedly enhances the anti-biofilm efficiency. Future studies are needed to assess FNTAP’s effects on long-term efficiency in controlling caries-related biofilms. FNTAP treatment could become an effective method to control biofilms associated with dental caries.

## Data Availability

The original contributions presented in this study are included in the article.

## Abbreviations

The following abbreviations are used in this manuscript:

BHI	Brain heart infusion
CFU	Colony forming units
DC	Direct current
DPBS	Dulbecco’s Phosphate-Buffered Saline
EPS	Extracellular polysaccharide
FNTAP	Fluoride non-thermal atmospheric plasma
MSA	Mitis Salivarius Agar
MTT	3-(4,5-Dimethyl-2-thiazolyl)-2,5-diphenyl-2H-tetrazolium bromide
NTAP	Non-thermal atmospheric plasma
OD600	Optical density at 600 nm
OES	Optical emission spectroscopy
sccm	Standard cubic centimeters per minute
*S. sanguinis*	*Streptococcus sanguinis*
*S. mutans*	*Streptococcus mutans*
TFE	1,1,1,2-tetrafluoroethane
